# Gastric cancer diagnosed after *Helicobacter pylori* eradication in diabetes mellitus patients

**DOI:** 10.1186/s12876-015-0377-0

**Published:** 2015-10-21

**Authors:** Kosuke Sakitani, Yoshihiro Hirata, Nobumi Suzuki, Satoki Shichijo, Ayako Yanai, Takako Serizawa, Kei Sakamoto, Masao Akanuma, Shin Maeda, Yutaka Yamaji, Yasuhiko Iwamoto, Shoji Kawazu, Kazuhiko Koike

**Affiliations:** The Institute for Adult Diseases, Asahi Life Foundation, 2-2-6 Bakuro-cho, Nihon-Bashi, Chuo-ku, Tokyo, 113-8655 Japan; Department of Gastroenterology, Graduate School of Medicine, The University of Tokyo, Tokyo, Japan; Gastroenterology Division, Yokohama City University Graduate School of Medicine, Yokohama, Japan

**Keywords:** Gastric cancer, *Helicobacter pylori* eradication, Diabetes mellitus

## Abstract

**Background:**

*Helicobacter pylori* infection is the most important risk factor for gastric cancer, for which eradication therapy is commonly performed. However, gastric cancer is sometimes discovered after successful eradication of *H. pylori*. Much evidence indicates that diabetes mellitus (DM) is a risk factor for gastric cancer. The incidence and characteristics of gastric cancer diagnosed after *H. pylori* eradication in DM patients remain to be determined.

**Methods:**

We followed the clinical course of patients who underwent *H. pylori* eradication therapy at our institution. Endoscopy was performed before and after eradication. We compared the incidence and clinical characteristics of gastric cancer arising in DM and non-DM patients.

**Results:**

In total, 965 patients who underwent successful eradication (518 DM and 447 non-DM patients) were followed-up for an average of 4.5 years. During the follow-up period, 21 gastric cancers were diagnosed (12 in DM patients and 9 in non-DM patients). The incidence of gastric cancer after eradication was not significantly different between DM and non-DM patients (0.485 and 0.482 %/year, respectively). There was no significant difference in the pathology, diameter, depth, location, or treatment of gastric cancer between patients with and without DM.

**Conclusion:**

The incidence and characteristics of gastric cancer occurring after *H. pylori* eradication were comparable between DM and non-DM patients.

## Background

Gastric cancer is a major cause of cancer death worldwide. Infection with *Helicobacter pylori* (*H. pylori*), first isolated by Warren and Marshall [1], together with the subsequent changes in the gastric mucosa, is the most important risk factor for gastric cancer [2–5]. Previous reports have suggested that *H. pylori* eradication reduces the incidence of gastric cancer [6–8]. Therefore, eradication therapy is now commonly performed. However, gastric cancer is occasionally discovered after successful *H. pylori* eradication, and investigations of the risk factors for, and characteristics of, gastric cancer after eradication have been conducted [9, 10].

The infection–inflammation–cancer axis is widely accepted, based in part on epidemiological and basic investigations of *H. pylori* [11]. On the other hand, evidence is accumulating that diseases associated with adult lifestyle factors—*e.g*., diabetes mellitus (DM)—enhance the risk of malignancies, including gastric cancer [12, 13]. Thus, it is possible that the presence of DM might affect the gastric carcinogenesis such as incidence and histological characteristics of gastric cancer after *H. pylori* eradication.

However, few studies have examined the inhibitory effect of *H. pylori* eradication therapy on gastric cancer in DM patients. In this study, we investigated the incidence and characteristics of gastric cancer diagnosed after *H. pylori* eradication in DM patients.

## Methods

### Patients

To evaluate the incidence and characteristics of gastric cancer diagnosed after *H. pylori* eradication in DM patients, 991 patients who underwent successful *H. pylori* eradication therapy at our institution between January 1996 and March 2013 and were followed up for over 6 months (the follow-up period was defined as the period from the day of commencement of eradication therapy to the day of the last endoscopy performed at our institution) were analyzed retrospectively. Then, we excluded patients who met the following criteria: i) those in whom endoscopy before eradication was not conducted at our institution (*n* = 18) and ii) those with a previous history of gastrectomy (*n* = 8). Therefore, a total of 965 patients were included in the analysis.

Patients were divided into DM and non-DM groups. DM was diagnosed according to the 2010 Japan Diabetes Society (JDS) criteria [14]. The diagnoses of hypertension (HT), hyperlipidemia (HL), and hyperuricemia (HU) were based on the need for medical treatment. The study design was approved by the Ethics Committee at the Institute for Adult Diseases, Asahi Life Foundation and conformed to the Declaration of Helsinki. The patients’ records were anonymized prior to analysis. Written informed consent was obtained from all participants.

### *H. pylori* eradication therapy

*H. pylori* infection was defined by a positive result for at least one of the following: rapid urease test (Helicocheck, Otsuka Pharmaceuticals, Tokyo, Japan), serological testing for anti-*H. pylori* IgG, ^13^C-urea urea breath test, or pathological analysis by hematoxylin and eosin or Giemsa staining. Patients in whom *H. pylori* infection was confirmed underwent eradication therapy. Patients in whom eradication therapy had failed received additional treatment: the first-line regimen comprised a proton pump inhibitor (PPI), amoxicillin, and clarithromycin [15]; the second-line regimen comprised a PPI, amoxicillin, and metronidazole; and the third-line regimen comprised a PPI, amoxicillin, and sitafloxacin [16]. The success or failure of eradication therapy was determined by a ^13^C-urea breath test performed at least 4 weeks after treatment.

### Endoscopy

At our institution, patients who receive eradication therapy are recommended to undergo esophagogastroduodenoscopy annually. The upper gastrointestinal endoscopies were performed by experienced endoscopists. Gastric mucosal atrophy was evaluated using the endoscopic scale described by Kimura and Takemoto [17], which has been reported to concord with the histological results [18]. Patients were classified into three groups according to their endoscopic atrophic gastritis grade: the mild (C-I and C-II), moderate (C-III and O-I), and severe atrophic gastritis (O-II and O-III) groups.

### Gastric cancer determination

Biopsy specimens were taken from the neoplastic lesion, and the final diagnosis of gastric cancer was based on the pathology results. Gastric cancers were classified pathologically according to Lauren’s classification as intestinal or diffuse type [19]. A lesion identified by endoscopy prior to eradication was not counted as gastric cancer after eradication in this study.

### Statistical analysis

All statistical analyses were performed using the JMP10 software (SAS Institute, Cary, NC, USA). Welch’s *t*-test was used to compare the means of continuous variables. Comparisons of nominal variables were performed by *χ*^2^ test or Fisher’s exact test, as appropriate. The incidence of gastric cancer in patients with and without DM was evaluated using the Kaplan–Meier method, and the statistical significance of the differences was evaluated by log-rank test. The diagnosis of gastric cancer was the primary endpoint, and data were censored at the last endoscopy. The risk of gastric cancer after eradication was assessed using Cox’s proportional-hazards model. Since no gastric cancer was detected in the mild atrophic gastritis group, this group served as the control. Odds ratios (OR) with 95 % confidence intervals (CI) were used as a measure of association and were adjusted using unconditional logistic regression models. A two-sided *p*-value of less than 0.05 was considered to indicate statistical significance.

## Results

### Baseline characteristics of the patients

The baseline characteristics of the patients are provided in Table 1. In total, 965 patients (729 males and 236 females, mean age 62.9 years, and mean body mass index (BMI) 23.2 kg/m^2^) underwent successful *H. pylori* eradication therapy as the first-, second-, and third-line regimens in 782 (81.0 %), 178 (18.4 %), and 5 (0.52 %) patients, respectively. The 965 patients comprised 518 DM patients (13 type 1 DM and 505 type 2 DM patients) and 447 non-DM patients. Patients were followed-up for up to 16.2 years (mean 4.5 years). In terms of the endoscopic atrophic gastritis grade, 173 (17.9 %), 422 (43.7 %), and 370 (38.3 %) patients were categorized as having mild, moderate, and severe atrophic gastritis, respectively.

**Table 1:** Characteristics of patients with and without DM who underwent *H. pylori* eradication therapy

Total (*n* = 965)	Non-DM (*n* = 447)	DM (*n* = 518)	*p*
Mean age (range), years	60.7 (27–84)	64.8 (30–86)	<0.0001*
Sex, *n* (%)			<0.0001*
Female	139 (31.1 %)	97 (18.7 %)	
Male	308 (68.9 %)	421 (81.3 %)	
Mean follow-up period (range), years	4.18 (0.54–13.2)	4.78 (0.68–16.2)	0.0006*
Mean endoscopy interval (range), years	1.37 (0.40–8.38)	1.61 (0.34–8.08)	<0.0001*
Mean body mass index (range), kg/m^2^	23.1 (14.8–34.9)	23.4 (15.9–32.3)	0.1452
Atrophic gastritis grade, *n* (%)			0.0011*
Mild	95 (21.3 %)	78 (15.1 %)	
Moderate	207 (46.3 %)	215 (41.5 %)	
Severe	145 (32.4 %)	225 (43.4 %)	
Hypertension, *n* (%)	99 (22.1 %)	244 (47.1 %)	<0.0001*
Hyperlipidemia, *n* (%)	89 (19.9 %)	220 (42.5 %)	<0.0001*
Hyperuricemia, *n* (%)	32 (7.16 %)	48 (9.27 %)	0.236

*DM* diabetes mellitus

*Statistically significant (*p* < 0.05)

Non-DM patients who underwent eradication therapy were significantly younger than DM patients (60.7 and 64.8 years, respectively). The mean follow-up period (4.18 years for non-DM patients and 4.78 years for DM patients) and mean endoscopy interval (1.37 years for non-DM patients and 1.61 years for DM patients) of the non-DM patients were significantly shorter than those of the DM patients. The mean BMI (23.1 kg/m^2^ for non-DM patients and 23.4 kg/m^2^ for DM patients) was not significantly different between non-DM and DM patients. The proportion of patients who received medications for HT and HL was higher in DM than in non-DM patients.

### Incidence and characteristics of gastric cancer after *H. pylori* eradication

During the follow-up period, 21 gastric cancers [9 in non-DM patients (0.482 %/year) and 12 in DM patients (0.485 %/year)] were diagnosed after *H. pylori* eradication (Table 2). No gastric cancer was found in the mild atrophic gastritis group, while 8 (38.1 %) gastric cancers were diagnosed in the moderate atrophic gastritis group and 13 (61.9 %) in the severe atrophic gastritis group. Among these 21 gastric cancers, there were no cardiac cancers. Eleven (52.4 %) gastric cancers had a depth of invasion limited to the mucosa; the remainder [10 (47.6 %)] exhibited submucosal or deeper invasion.

**Table 2:** Characteristics of gastric cancer in patients with and without DM after *H. pylori* eradication therapy

Total (*n* = 21)	Non-DM (*n* = 9)	DM (*n* = 12)	*p*
Mean age (range), years	69.4 (56–80)	70.6 (60–78)	0.7137
Sex, *n* (%)			0.5533
Female	2 (22.2 %)	1 (8.33 %)	
Male	7 (77.8 %)	11 (91.7 %)	
Mean follow up period (range), years	4.59 (1.03–11.8)	3.95 (0.95–7.15)	0.6990
Mean endoscopy interval (range), years	1.93 (0.99–4.06)	1.62 (0.70–2.95)	0.2527
Mean body mass index (range), kg/m^2^	23.4 (19.5–27.5)	22.6 (19.0–30.1)	0.5534
Atrophic gastritis, *n* (%)			0.2030
Mild	0	0	
Moderate	5 (55.6 %)	3 (25.0 %)	
Severe	4 (44.4 %)	9 (75.0 %)	
Pathology, *n* (%)			0.3383
Intestinal type	8 (88.9 %)	8 (66.7 %)	
Diffuse type	1 (11.1 %)	4 (33.3 %)	
Diameter, mm	22.1 (3–70)	25.0 (5–60)	0.7543
Depth, *n* (%)			0.2562
Mucosa	6 (66.7 %)	5 (41.7 %)	
Submucosa and deeper	3 (33.3 %)	7 (58.3 %)	
Location, *n* (%)			0.7332
Upper third	3 (33.3 %)	2 (16.7 %)	
Middle third	3 (33.3 %)	5 (41.7 %)	
Lower third	3 (33.3 %)	5 (41.7 %)	
Treatment, *n* (%)			0.3869
Endoscopic	6 (66.7 %)	5 (41.7 %)	
Surgery	3 (33.3 %)	7 (58.3 %)	

*DM* diabetes mellitus

The mean age (69.4 years for non-DM patients and 70.6 years for DM patients), the proportion of males (77.8 % for non-DM patients and 91.7 % for DM patients), mean follow-up duration (4.59 years for non-DM patients and 3.95 years for DM patients), mean endoscopy interval (1.93 years for non-DM patients and 1.62 years for DM patients), mean BMI (23.4 kg/m^2^ for non-DM patients and 22.6 kg/m^2^ for DM patients) and atrophic gastritis grade were not significantly different between the gastric cancer patients with versus without DM.

In total, 16 (76.2 %) intestinal-type gastric cancer and 5 (23.8 %) diffuse-type gastric cancer cases were diagnosed. Among the non-DM gastric cancer patients, 88.9 % (8/9) were of the intestinal type, compared with 66.7 % (8/12) of the DM gastric cancer patients. Regarding gastric cancer treatment modalities, 11 (52.4 %) patients underwent curative endoscopic treatment. The remaining 10 (47.6 %) patients received curative surgical treatment. There were no significant differences in the pathology, diameter (22.1 mm for non-DM patients and 25.0 mm for DM patients), depth, location, or treatment of the gastric cancers between patients with and without DM.

**Fig. 1:**
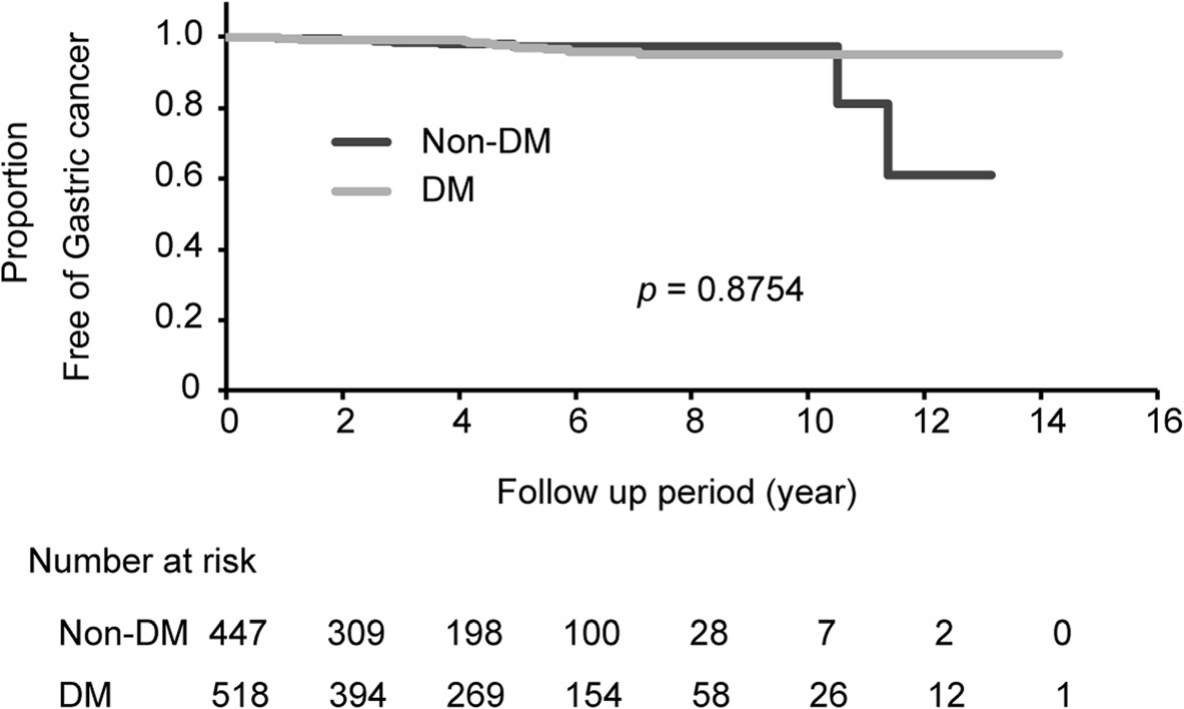
Kaplan–Meier analysis of the proportion of patients with and without diabetes mellitus (DM) free from gastric cancer after *H. pylori* eradication therapy. Gastric cancer was diagnosed in nine non-DM patients and 12 DM patients during the follow-up period (*p* =0.8754, log-rank test)

Kaplan–Meier analysis of the proportions of patients free of gastric cancer after *H. pylori* eradication with and without DM is shown in Fig. 1. No significant difference was found in the incidence of gastric cancer after eradication between non-DM and DM patients by log-rank test (*p* = 0.8754).

### Risk factors for gastric cancer after *H. pylori* eradication

We next analyzed the risk factors for gastric cancer after *H. pylori* eradication using Cox’s proportional hazards model (Table 3). Age, sex, BMI, DM, HT, HL, and HU were not found to be risk factors for gastric cancer after *H. pylori* eradication in either univariate or multivariate analysis. Severe atrophic gastritis before eradication was identified as a significant risk factor for gastric cancer after *H. pylori* eradication in the multivariate analysis (OR 2.56, 95 % CI 1.03–6.77, *p* = 0.0424), in accordance with a previous report [9].

**Table 3:** Cox’s proportional hazards model for factors associated with the incidence of gastric cancer after *H. pylori* eradication

Factor	Univariate	*p*	Multivariate	*p*
	OR (95 % CI)		OR (95 % CI)	
Age (years)	1.01 (0.96–1.06)	0.6248	1.00 (0.94–1.05)	0.9446
Sex				
Female	1			
Male	1.82 (0.61–7.78)	0.3030	2.13 (0.67–9.46)	0.1914
Body mass index kg/m2) m^2^)	1.02 (0.83–1.13)	0.7880	1.01 (0.83–1.15)	0.8264
Diabetes mellitus (DM)				
Non-DM	1			
DM	0.93 (0.39–2.30)	0.8756	0.64 (0.25–1.66)	0.3530
Hypertension (HT)				
Non-HT	1			
HT	1.40 (0.57–3.31)	0.4459	1.36 (0.53–3.36)	0.4985
Hyperlipidemia (HL)				
Non-HL	1			
HL	1.15 (0.43–2.83)	0.7600	1.17 (0.42–2.99)	0.7485
Hyperuricemia (HU)				
Non-HU	1			
HU	0.62 (0.03–3.05)	0.6295	0.43 (0.02–2.24)	0.3811
Atrophic gastritis				
Mild-Moderate	1			
Severe	2.63 (1.10–6.67)	0.0280*	2.56 (1.03–6.77)	0.0424*

*OR* odds ratio; *CI* confidence interval

*Statistically significant (*p* < 0.05)

## Discussion

In this study, we aimed to elucidate the incidence and characteristics of gastric cancer after *H. pylori* eradication therapy in DM patients. Our results indicated that the incidence and characteristics of gastric cancer developing after *H. pylori* eradication therapy were not significantly different between DM and non-DM patients. The incidence of gastric cancer after eradication was 0.48 %/year and the mean age of the patients 62 years. The incidence and mean age are in agreement with those reported by Ogura et al. (0.46 %/year and 62 years, respectively) [6]. Kamada et al. reported that severe mucosal atrophy in the corpus is a risk factor for gastric cancer after eradication [9]. This study also showed that severe atrophic gastritis before eradication is the risk for gastric cancer suggesting follow-up endoscopy of patients with severe atrophic gastritis is necessary.

The prevalence of DM is rapidly increasing, and several studies have focused on the interaction of DM with *H. pylori* infection and the gastric cancer axis [20–22]. The prevalence of *H. pylori* infection in DM patients is reportedly higher than that in non-DM patients [23]. Sekikawa et al. reported that open-type atrophic gastritis is found more frequently in DM patients than in non-DM patients (33.4 % (50/148) and 21.1 % (274/1301), respectively); moreover, the presence of DM increased the risk of development of early gastric cancer. In the present study, the presence of DM did not increase the incidence of gastric cancer after *H. pylori* eradication therapy. It is possible that DM acts synergistically with consistent *H. pylori* infection to increase the risk of gastric cancer, as reported previously [24], and that the presence of DM after *H. pylori* eradication therapy might not enhance the risk of gastric cancer.

Other possible risk factors for gastric cancer (such as smoking and high salt intake) have been reported to increase gastric cancer risk synergistically with *H. pylori* infection [25, 26]. A prospective study conducted in Japan (the Hisayama study) found that the combination of smoking and *H. pylori* infection increased the risk of gastric cancer more than did smoking or *H. pylori* infection alone [25]. The Hisayama study also found a significant association between high salt intake and gastric cancer in a population with atrophic gastritis and *H. pylori* infection [26].

In the present study, the pathological features of gastric cancer after eradication were not significantly different between DM and non-DM patients. This finding is in line with a previous report that there is no significant difference in the histopathological differentiation of gastric cancer in patients with versus without DM [27]. The proportion of those with intestinal-type gastric cancer after eradication (76.2 %, 16/21) in this study is in agreement with that reported by Kamada et al. (75.0 %, 15/20) [9].

There were several limitations to our study. First, it was a retrospective cohort design; therefore, further prospective and longer-term studies are required. Second, several possible risk factors for gastric cancer—such as smoking, alcohol intake, dietary habits, family history and *H. pylori* virulence factors—were not evaluated. Regarding lifestyle factors, a previous report showed that smoking and drinking alcohol were not associated with the development of gastric cancer after *H. pylori* eradication therapy [28].

## Conclusion

Our findings indicated that the incidence and characteristics of gastric cancer after *H. pylori* eradication therapy were comparable between DM and non-DM patients.

## Abbreviations

DM: Diabetes mellitus
